# DCE-MRI biomarkers of tumour heterogeneity predict CRC liver metastasis shrinkage following bevacizumab and FOLFOX-6

**DOI:** 10.1038/bjc.2011.191

**Published:** 2011-06-14

**Authors:** J P B O'Connor, C J Rose, A Jackson, Y Watson, S Cheung, F Maders, B J Whitcher, C Roberts, G A Buonaccorsi, G Thompson, A R Clamp, G C Jayson, G J M Parker

**Affiliations:** 1 Imaging Science, Proteomics and Genomics Research Group, School of Cancer and Enabling Sciences, University of Manchester, Manchester Academic Health Sciences Centre, Oxford Road, Manchester M13 9PT, UK; 2 Department of Radiology Christie Hospital, Wilmslow Road, Manchester M20 4BX, UK; 3 Clinical Imaging Centre, GlaxoSmithKline, Hammersmith Hospital, Imperial College London, Du Cane Road, London W12 0HS, UK; 4 Cancer Research UK Department of Medical Oncology, Christie Hospital, Wilmslow Road, Manchester M20 4BX, UK

**Keywords:** angiogenesis, biomarker, heterogeneity, MRI, outcome, personalised medicine

## Abstract

**Background:**

There is limited evidence that imaging biomarkers can predict subsequent response to therapy. Such prognostic and/or predictive biomarkers would facilitate development of personalised medicine. We hypothesised that pre-treatment measurement of the heterogeneity of tumour vascular enhancement could predict clinical outcome following combination anti-angiogenic and cytotoxic chemotherapy in colorectal cancer (CRC) liver metastases.

**Methods:**

Ten patients with 26 CRC liver metastases had two dynamic contrast-enhanced MRI (DCE-MRI) examinations before starting first-line bevacizumab and FOLFOX-6. Pre-treatment biomarkers of tumour microvasculature were computed and a regression analysis was performed against the post-treatment change in tumour volume after five cycles of therapy. The ability of the resulting linear model to predict tumour shrinkage was evaluated using leave-one-out validation. Robustness to inter-visit variation was investigated using data from a second baseline scan.

**Results:**

In all, 86% of the variance in post-treatment tumour shrinkage was explained by the median extravascular extracellular volume (*v*_e_), tumour enhancing fraction (*E*_F_), and microvascular uniformity (assessed with the fractal measure box dimension, *d*_0_) (*R*^2^=0.86, *P*<0.00005). Other variables, including baseline volume were not statistically significant. Median prediction error was 12%. Equivalent results were obtained from the second scan.

**Conclusion:**

Traditional image analyses may over-simplify tumour biology. Measuring microvascular heterogeneity may yield important prognostic and/or predictive biomarkers.

There is considerable interest in developing pre-treatment biomarkers of microvascular structure and function that predict subsequent therapeutic response (O'Connor *et al*, 2008). Development and validation of such biomarkers will be essential if personalised therapy is to become a reality (Meyer *et al*, 2009).

Tumour size is an important factor in staging some solid tumours, selecting treatment options, and in predicting clinical outcome (Edge *et al*, 2010). However, for some solid tumours, size has little relevance to tumour stage and the link between pre-treatment tumour size and outcome is complex, with no clear relationship between the two (Grigsby *et al*, 1999; Foulkes *et al*, 2008; Klatte *et al*, 2008).

Tumour function may also predict outcome, particularly in the setting of novel adjuvant therapies. Techniques such as ^18^F-fluorodeoxyglucose positron emission tomography (^18^F-FDG-PET) and dynamic contrast-enhanced MRI (DCE-MRI) offer the opportunity to study tumour pathophysiology (O'Connor *et al*, 2008). For example, simple summary values such as high baseline ^18^FDG-PET standardised uptake value (Dimitrakopoulou-Strauss *et al*, 2004; de Geus-Oei *et al*, 2006) and high baseline DCE-MRI volume transfer constant (*K*^trans^) (George *et al*, 2001) before therapy have shown statistically significant relationships with beneficial clinical outcome following various cytotoxic treatments in patients with colorectal cancer (CRC). However, current evidence that these biomarkers accurately predict clinical outcome is limited (O'Connor *et al*, 2007a; Kinahan *et al*, 2009).

This relative lack of success has fuelled interest in alternative biomarkers of microvascular structure and function that may better serve as predictive and/or prognostic biomarkers. There is emerging evidence that tumours are biologically complex structures exhibiting marked spatial variation in angiogenesis (Kumar *et al*, 1998; Eberhard *et al*, 2000), hypoxia (Picchio *et al*, 2008), cell death (Gonzalez-Garcia *et al*, 2002), and glucose metabolism (Schroeder *et al*, 2005). The presence and degree of heterogeneity may be an important determinant of cancer metastatic potential (Fidler, 1987) and response to therapy (Casanovas *et al*, 2005). Despite this, measurement of tumour heterogeneity is largely ignored in radiological practice.

Previous imaging studies have provided evidence of a relationship between the spatial heterogeneity of image biomarkers of the tumour vasculature (such as *T*_1_ signal change, *K*^trans^, blood volume, and Hounsfield units) and therapeutic response (Jackson *et al*, 2007). Several differing approaches have been studied, including histogram analysis (Chang *et al*, 2004) and measuring the proportion of the tumour that enhances (O'Connor *et al*, 2007b). Both of these approaches summarise the distribution of tumour functional properties but ignore the spatial location of individual tumour voxels (and their vascular, metabolic, or other features) and the relationship of one voxel to another within a lesion. Alternative approaches such as texture (El Naqa *et al*, 2009) or fractal analysis (Dzik-Jurasz *et al*, 2004; Rose *et al*, 2007; Goh *et al*, 2009; Alic *et al*, 2011) quantify the overall spatial complexity of a tissue and retain information regarding the spatial arrangement of voxels within a tumour. In this study, we tested the hypothesis that tumour measurements of microvascular function and heterogeneity computed from pre-treatment DCE-MRI data would predict tumour shrinkage following combination therapy with anti-angiogenic and cytotoxic chemotherapy.

## Materials and methods

### Study design

Retrospective analysis was performed on DCE-MRI data collected between July 2006 and October 2007 in 10 patients with CRC liver metastases. Patients were originally recruited for an investigator led study that examined the temporal action of bevacizumab during a single cycle of therapy (O'Connor *et al*, 2009). Ethical approval was granted by the local Research Ethics Committee and informed consent was obtained. All patients received single agent 10 mg kg^–1^ bevacizumab (cycle 1) followed every 2 weeks by 10 mg kg^–1^ bevacizumab plus FOLFOX-6 (oxaliplatin/5FU/leucovorin) for 2 months (cycles 2–5) as first-line treatment.

### Patient recruitment

Patients with histologically proven primary epithelial CRC, aged ⩾18 years, with an Eastern Cooperative Oncology Group score between 0 and 2, and life expectancy of at least 3 months were eligible. Enrolment of patients who required first-line treatment for metastatic disease was consecutive. Inclusion criteria were presence of a measurable lesion ⩾2 cm on previous imaging; adequate liver, renal, and haematologic function; normal coagulation (prothrombin time and activated partial thromboplastin time); normal ECG.

Exclusion criteria were previous treatment with vascular endothelial growth factor (VEGF) inhibitors or cytotoxic chemotherapy; contraindications to VEGF inhibitors; exposure to any other investigational drug (within the last 4 weeks) or concurrent therapy likely to influence the vasculature on imaging; pregnant or breast-feeding women; previous clinically significant haemorrhage, thrombosis, or cardiovascular disease within the last 6 months; proteinuria; contraindication to MRI.

### MRI data acquisition

Patients were examined on a 1.5-T Philips Intera system (Philips Medical Systems, Best, The Netherlands). Each patient was scanned twice before treatment, to allow measurement reliability to be assessed. In one patient (with three tumours), data were only available from one pre-treatment scan. The field of view (FOV) was centred on the liver. In each examination, *T*_1_-weighted fast field echo images (TR=10 ms, TE=4.6 ms, *α*=15°) and *T*_2_-weighted single shot turbo spin echo images (TR=606.5 ms, TE=80 ms, *α*=90°) were acquired. Both sequences employed FOV 375 × 375 mm^2^, matrix 256 × 256 with a 4-mm slice thickness.

For the DCE-MRI series, 75 3D axial volumes were acquired consecutively (TR=4.0 ms, TE=0.82 ms, *α*=20°, one signal average, FOV of 375 × 375 mm^2^, matrix 128 × 128; in-plane voxel size 2.93 × 2.93 mm^2^) following calculation of baseline *T*_1_ using the variable flip angle method (Fram *et al*, 1987) (*α*=2°/10°/20° four signal averages; identical TR, TE, imaging matrix, and slice thickness). Temporal resolution was 4.97 s. On the sixth dynamic time point, 0.1 mmol kg^–1^ of *Gadodiamide* (Omniscan GE Medical Systems, Amersham, UK) was administered intravenously through a Spectris MR power injector (Medrad Inc., Indianola, PA, USA) at 3 ml s^–1^, followed by a saline flush. Slice thickness was 4 mm for small target lesions or 8 mm for larger lesions, giving superior–inferior coverage of either 100 or 200 mm. Images were acquired during gentle free breathing.

### Calculation of tumour volume and summary DCE-MRI statistics

Quality control was applied to reduce error in all image parameters. The impact of motion was assessed and tumours for which parameter estimates would be unreliable were rejected. The level of bulk motion was assessed for each tumour by first extracting an averaged time series plot for each tumour region of interest (ROI) on each slice in the imaging volume and then by visual assessment of the dynamic time series images. In- and through-plane motion was investigated and a categorical score was assigned for each tumour based on the evaluations of bulk motion (slight motion=1, moderate motion=2, significant motion=3, and severe motion=4). Tumours with a motion assessment score of 3 or 4 were excluded.

Three-dimensional ROIs were defined on coregistered high-resolution *T*_1_- and *T*_2_-weighted sequences. In some patients, multiple lesions were defined. To quantify microvascular characteristics, the extended Tofts version of the Kety model (Tofts, 1997) was fitted to the DCE-MRI time series at each enhancing tumour voxel (see below) using in-house software, as given by 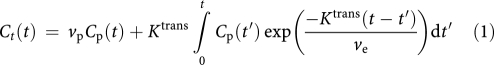 where *C*_*t*_(*t*) is the concentration of contrast agent at time *t* in each voxel and *C*_p_(*t*) is the concentration of contrast agent in the arterial blood plasma (i.e., the arterial input function, which was determined using a previously published population AIF technique (Parker *et al*, 2006)). The extended Tofts model is only valid in tumour regions in which there is a measurable signal change due to the presence of contrast agent. To identify those voxels, a previously published method was used in which pre- and post-injection concentration values are statistically compared (O'Connor *et al*, 2009).

**Figure 1:**
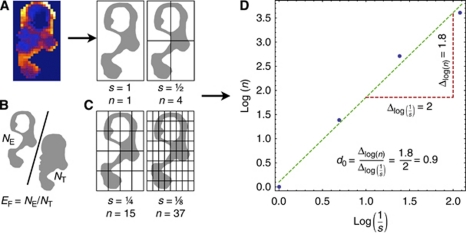
Derivation of a thresholded parameter map to enable calculation of enhancing fraction (*E*_F_) and box dimension (*d*_0_). (**A**) *K*^trans^ map across a single slice within a CRC liver metastasis shows marked spatial heterogeneity. (**B**) A criterion is applied to the contrast agent concentration time series to identify enhancing voxels (*N*_E_) and the resultant map is shown. *E*_F_ is calculated as the ratio of *N*_E_ to the number of tumour voxels (*N*_T_). (**C**) A box surrounding the object defined by the enhancing voxels is successively divided, defining a range of scales (*s*) at which the number of boxes containing a part of the object is counted (*n*). (**D**) *d*_0_ is the slope of the line of best fit through the points (log *n*, log 1/*s*) and quantifies the space filling properties of the parameter map.

Voxel-wise analysis was performed allowing estimates of the median bulk transfer coefficient (*K*^trans^; units min^−1^; Figure 1A), mean fractional plasma volume (*v*_p_; unitless) and median fractional volume of the extravascular extracellular space (*v*_e_; unitless).

### Calculation of heterogeneity statistics

The following three types of heterogeneity statistic were derived:

1. Enhancing fraction (*E*_F_, defined as the ratio of the number of enhancing voxels to total tumour voxels; *E*_F_=*N*_E_/*N*_T_) was calculated to characterise the overall level of tumour perfusion (Figure 1B). Voxels with pre-contrast and post-contrast agent time series that had significantly different distributions (where *P*<0.05 on Mann–Whitney Wilcoxon rank-sum test) were classified as enhancing (O'Connor *et al*, 2007b).
2. Standard deviations of the voxel-wise *K*^trans^, *v*_e_, and *v*_p_ measurements were calculated for the enhancing portion of each tumour.
3. Fractal summaries (measures of microvascular structure and function that consider spatial information) were derived from the DCE-MRI data. The calculation of one of these fractal measures, box dimension (*d*_0_), is illustrated (Figures 1C and D), where a binary image corresponding to enhancing tumour voxels is iteratively subsampled by a factor of two to define binary images at a range of scales; at each scale, the enhancing voxels are counted and *d*_0_ is calculated as the rate of change in the number of enhancing voxels with respect to scale (with both quantities considered on logarithmic scales). In addition, fractal measures based on the information dimension (*d*_1_) and correlation dimension (*d*_2_) were calculated. These latter metrics retain magnitude values of *K*^trans^, *v*_e_, and *v*_p_ and are described in detail elsewhere (Rose *et al*, 2009).

### Evaluation of tumour shrinkage

X-ray computed tomography (CT) examination of the abdomen and pelvis was performed at baseline within 72 h of the baseline MRI and at the end of cycle 5 (EC5) to evaluate clinical response. Patients were imaged on a LightSpeed Plus CT scanner (GE Medical Systems), with typical clinical helical acquisition variables (tube voltage 120 kV, tube current 40 mA). Images were acquired following intravenous injection of 200 ml Omnipaque-140 (GE Medical Systems) and reformatted to produce contiguous 5 mm slices with no overlap. Tumour volumes were measured (in mm^3^) and the remaining tumour volume (%) from baseline to EC5 was calculated by comparison with the pre-treatment tumour volume.

### Statistical analysis

Percentage of remaining tumour volume at EC5 was modelled as a linear function of the pre-treatment summary statistics described above. Before modelling, variables were transformed as appropriate to improve linearity (e.g., by taking logarithms). In a preliminary analysis, a mixed-effects model was used to explore potential within-patient clustering since some patients had more than one tumour. However, no statistically significant evidence for such effects was found. Subsequently, tumours were treated independently.

Stepwise errors-in-variables regression was used to model the percentage of remaining tumour volume based on the pre-treatment volume, summary DCE-MRI statistics, and heterogeneity statistics using data from the first pre-treatment scan. The regression was repeated using the data from the second scan to investigate robustness to inter-visit variation. In the above analyses, the missing data for one patient's first pre-treatment scan (three tumours) were dealt with using list-wise deletion or imputation (from the second scan) as appropriate, with the aim of maximising the amount of data available, while minimising bias.

The ability of the linear model to predict tumour response was evaluated using two leave-one-out analyses. In the first analysis, each *tumour* was left out in turn; the coefficients on each variable were computed—by applying errors-in-variables regression to the left-in tumours' data—and used to predict the response of the left-out tumour. In the second analysis, the data for each *patient* was left out in turn (allowing us to further investigate potential intra-patient clustering effects); the coefficients on each variable were computed—by applying errors-in-variables regression to the left-in patients' data—and used to predict the responses for the tumours in the left-out patients.

Prediction error was quantified using the absolute difference between the actual and predicted percentage of remaining tumour volume. A cumulative distribution function (CDF) of prediction error was plotted for each leave-one-out analysis; a CDF permits estimation of the proportion of predictions that would be expected to be less than or equal to a given prediction error. Bland–Altman plots were formed to assess the agreement between actual and predicted percentage of the remaining tumour volume.

Statistical modelling was performed using Stata/IC version 10.1 (Stata Corporation, College Station, TX, USA) and leave-one-out analysis was performed using Mathematica version 7.0.1 (Wolfram Research, Champaign, IL, USA).

## Results

The mean patient age was 68.3 years (range 61–78 years; eight males; two females). All patients completed therapy to EC5. Two patients achieved partial responses; seven had stable disease; one had disease progression by RECIST 1.0 criteria. In all, 26 tumours were identified in the 10 patients (mean 2.6, median 2.5).

**Figure 2:**
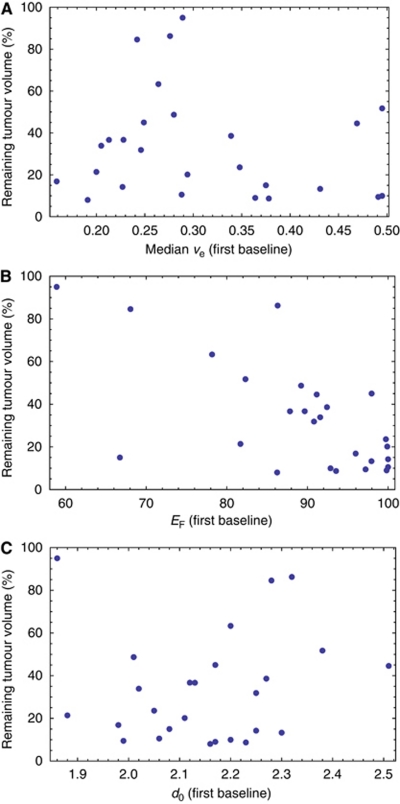
Scatter plots showing the relationship between (**A**) median *v*_e_, (**B**) *E*_F_, and (**C**) *d*_0_ and remaining tumour volume (%).

The final errors-in-variables regression analysis modelled tumour response in terms of the following pre-treatment biomarkers: median *v*_e_, *E*_F_, and *d*_0_ (details in Table 1). This model explained 86% of the variance in tumour response (95% confidence interval 77–94%). When the regression was repeated for the second pre-treatment scan data, no significant differences between the coefficients estimated for each variable, or between the *R*^2^ values, were found; however, median *v*_e_ was not quite significant within this second model (*P*=0.07). This suggests that the identified variables and the underlying model are robust to typical inter-visit variation. Scatter plots showing the relationships between the retained variables and percentage remaining tumour volume are provided in Figure 2.

**Figure 3:**
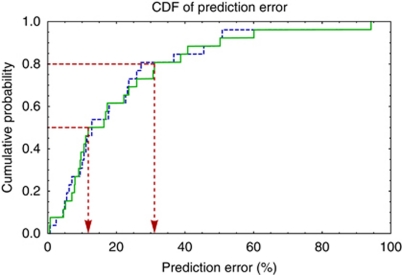
Cumulative distribution functions of prediction error for the leave-one-*tumour*-out (dashed blue) and leave-one-*patient*-out (solid green) analyses. Red lines show prediction error for 50% and 80% of all cases.

The results of the two leave-one-out analyses suggest that tumour response can be predicted with an error of no more than 12% in 50% of cases, and with an error of no more than 31% in 80% of cases (Figure 3). No difference was observed between the leave-one-out analysis that treated tumours independently and that grouped tumours at the patient level, indicating no evidence for intra-patient clustering.

**Figure 4:**
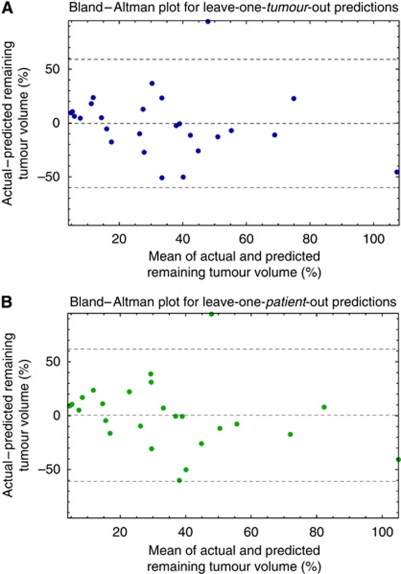
Bland–Altman plots for (**A**) leave-one-*tumour*-out and (**B**) leave-one-*patient*-out predictions, showing the mean difference between the actual and predicted changes in volume, and that mean ±2 s.d. of the differences.

The Bland–Altman plots for the leave-one-tumour- and leave-one-patient-out predictions were very similar, with the differences (vertical axes) having almost identical mean (−0.04%) and standard deviation (30%) (Figure 4). In one tumour, the remaining tumour volume was predicted particularly poorly (the model dramatically underestimates the actual change). There was no statistically significant relationship between the means and differences.

## Discussion

In this study, we have investigated the relative value of pre-treatment biomarkers of the microvasculature in explaining the percentage of remaining tumour volume resulting from combined bevacizumab and FOLFOX-6 therapy. We hypothesised that tumour size change *could* be explained by baseline image data, but that simple measures of size or function (used individually) may lack predictive power.

In this data set, 86% of the variance in the outcome measure (percentage remaining tumour volume EC5) was explained by combining various pre-treatment imaging biomarkers. Importantly, robustness to inter-visit variation was validated by a second data set derived from the same tumours. Of note, pre-treatment tumour volume was not found to be a statistically significant determinant of subsequent change in tumour volume following treatment. However, three variables (*v*_e_, *E*_F_, and *d*_0_) were statistically significant within the model and provide complementary types of information about the tumour environment. These results are congruent with other studies, which report that multi-parametric image analyses may be better at predicting clinical outcome (Dimitrakopoulou-Strauss *et al*, 2004, 2010), compared with more traditional analyses based around a single parameter.

Median *v*_e_ is an estimate of the extracellular extravascular space affected by factors including cell size, number, and packing density. It also represents a direct estimate of the distribution space to which a contrast agent or drug can be delivered. Different studies have reported variably either decrease or increase in median *v*_e_ in small numbers of patients following anti-vascular therapy. No study has reported how this parameter may relate to a measure of clinical outcome. In this study, high median *v*_e_ was associated with greater tumour shrinkage, which may indicate the potential for greater extravasations of chemotherapy and bevacizumab into the extracellular extravascular space.

Two measures of tumour vascular heterogeneity – *E*_F_ and *d*_0_ – were also statistically significant in this study. *E*_F_ is the ratio of enhancing tumour voxels to overall tumour voxels. As such, it is a crude indicator of heterogeneity by quantifying the proportion of a tumour that has demonstrable delivery and retention of contrast agent. For example, a tumour with cystic or necrotic components has a lower *E*_F_ than a predominantly solid lesion. The parameter is repeatable with a low coefficient of variation (O'Connor *et al*, 2009) and is sensitive to the therapeutic effect of anti-angiogenic and anti-vascular compounds (Galbraith *et al*, 2003; Robinson *et al*, 2003; O'Connor *et al*, 2009). In this study, high *E*_F_ was associated with better tumour response. This is consistent with identifying tumours with a high proportion of well-perfused tissue that receive substantial penetration of systemically administered agents.

Previous studies have reported that high baseline *E*_F_ predicted poor response to cytotoxic chemotherapy in epithelial ovarian cancer (O'Connor *et al*, 2007b) and radiotherapy in cervical cancer (Donaldson *et al*, 2010), attributed to a high *E*_F_ representing a greater amount of neo-angiogenic tumour tissue. This apparent discrepancy may reflect the fact that *E*_F_ is a non-specific marker that is influenced by several physiological correlates including flow, permeability, vascular volume, extravascular leakage space, and interstitial pressure. *E*_F_ may therefore, be best interpreted relative to normal tissue values rather than absolute values that may indicate prognosis. The type of treatment employed and method of calculating enhancement also vary between studies and alter the relative meaning of a high or low *E*_F_.

Enhancing fraction and *v*_e_ disregard spatial information; two parameter maps with identical *E*_F_ or *v*_e_ values can have completely different spatial distributions of either parameter (Rose *et al*, 2009). For this reason, there has been interest in developing metrics such as box dimension that quantify the spatial heterogeneity present within parametric imaging maps of tumours (Goh *et al*, 2009). In this study, low box dimension (*d*_0_) was associated with better tumour response. This parameter can be low if there are few enhancing voxels or if enhancing voxels are non-uniformly distributed. The proportion of enhancing voxels is already captured in *E*_F_ but *d*_0_ is also significant, implying that the spatial arrangement of enhancing tissue within a tumour (and therefore the uniformity of drug delivery) is important. As used here, *d*_0_ reflects not only microvascular uniformity but also depends on tumour shape. Our data are comparable to a study of rectal carcinoma treated by cytotoxic chemotherapy, where a similar parameter calculated from pre-treatment thresholded single slice parameter maps for area under the Δ*R*_2_^*^ curve (where *R*_2_^*^=1/*T*_2_^*^) predicted tumour regression after 8 weeks of therapy (Dzik-Jurasz *et al*, 2004) and a study of limb sarcomas where fractal dimension distinguished responders from non-responders (Alic *et al*, 2011).

The CDFs presented in Figure 3 show that in general, percentage remaining tumour volume can be predicted with relatively little error in this particular clinical scenario. This application may be extremely useful for the selection of patients likely to benefit from expensive novel therapies, as it may be possible to identify patients whose tumours that are more or less likely to respond to therapy. The little difference between the CDFs for the leave-one-tumour- and leave-one-patient-out analyses, suggest that the ability of the variables identified to predict tumour response cannot easily be explained by the influence of patients with multiple tumours. The resulting model explains a large proportion (86%) of the total variance in tumour response and allows tumour response to be predicted with excellent accuracy in the majority of cases. However, while the variables were highly significant within the model, the confidence intervals on their coefficients are wide; a larger sample would be required to estimate these coefficients with more confidence. The model was also robust to inter-visit variation, since the same variables were significant (with the exception of median *v*_e_), and there were no significant differences between the coefficient or *R*^2^ values – providing internal validation.

Our study has four main limitations. First, it was retrospective. Second, the imaging parameters used require significant post-processing effort to obtain and do not have accepted standardisation. Third, while the biomarkers identified appear to predict shrinkage, they require testing against survival in a larger study. Fourth, respiratory or other patient motion can complicate any image analysis, particularly those performed on a per voxel basis (Orton *et al*, 2009). Image registration can be used to salvage motion-corrupted data, but requires additional post-processing work, limiting its applicability in clinical settings (Buonaccorsi *et al*, 2006, 2007). Our results demonstrate that tumour shrinkage can be predicted even in the presence of typical patient motion (however, note that we did reject data that were corrupted by very significant motion). Future work should determine if image registration offers any advantage in the context of predicting tumour shrinkage or survival. Finally, in addition to seeking to improve prediction accuracy, future work should also investigate the use of imaging (or other) information to identify tumours for which the kind of model proposed here would perform poorly.

In conclusion, these data provide preliminary evidence that a combination of pre-treatment measures derived from DCE-MRI parameter maps may predict tumour shrinkage in response to combined bevacizumab and cytotoxic chemotherapy in CRC liver metastases, with relatively low error. Although the applicability of these results to other tumours types and metastatic disease in other organs cannot be inferred from the data presented, this type of approach may have value in determining personalised tumour therapy regimes and patient selection. These results encourage the further evaluation of image heterogeneity in cancer studies using MRI, PET, and other techniques, to explore whether similar findings are seen in alternative combinations of patient group, therapy, and imaging technique.

## Figures and Tables

**Table 1:** Result of the errors-in-variables regression shows: the model's F statistic, *P*, and *R*^2^ values

				F_3, 22_	25.86
				*P*	⩽0.00005
				*R* ^2^	0.86
**Variable**	**Coefficient**	***T***	***P*>∣*t*∣**	**95% CI on coefficient**	
*v*_e_	−147.08	−3.37	0.003	−237.49	−56.67
*E*_F_	−2.35	−8.46	⩽0.0005	−2.93	−1.78
*d*_0_	156.10	4.04	0.001	75.91	236.30
Constant	−47.19	−0.68	0.506	−191.83	97.45

Abbreviations: *d*_0_=fractal measure box dimension; *E*_F_=enhancing fraction; *v*_e_=median extravascular extracellular space volume.

The variables listed were significant in the final model (corresponding coefficients, *t* statistics, *P* values, and 95% confidence intervals (CIs) are provided). The constant term in the linear model is also included.
